# Development of a SMCs model from HGPS-iPS and proofs of principle

**DOI:** 10.1186/1750-1172-10-S2-O3

**Published:** 2015-11-11

**Authors:** Lino Ferreira

**Affiliations:** 1 Center of Neurosciences and Cell Biology, University of Coimbra, Portugal

## 

HGPS is a rare, progressive aging disease in children that leads to premature death. Vascular smooth muscle cells (SMCs) are the most affected cells in HGPS patients, although the reason for such sensitivity remains poorly understood. Induced pluripotent stem cells (iPSCs) offer an unlimited source of SMCs to study the disease. iPSCs are also an important tool to study the molecular mechanisms of the disease from a developmental point of view. In this work, we study the reasons of HGPS-SMCs vulnerability using iPSCs obtained from HGPS fibroblast patients. We have evaluated the differentiation profile of HGPS-iPSCs and normal iPSCs into SMCs. We showed that HGPS-iPSC SMCs shared similar features observed on progerin-expressing cells. We have identified and characterize drugs that prevent SMC loss. Our findings open new opportunities for the treatment of HGPS disease and diseases related to vascular ageing.

